# Supplementary material for: The interactome of intact mitochondria by cross-linking mass spectrometry provides evidence for coexisting respiratory supercomplexes
Source: Mol Cell Proteomics. 2017 Dec 8;17(2):216–32. doi: 10.1074/mcp.RA117.000470 (PMC5795388; doi:10.1074/mcp.RA117.000470)
Supplement: Supplemental Data [file supp_RA117.000470_133922_0_supp_23978_fzffwf.zip › spectra_annotation/mito_DR_spectra_annotation/144-1-10-1-5-1.pdf]

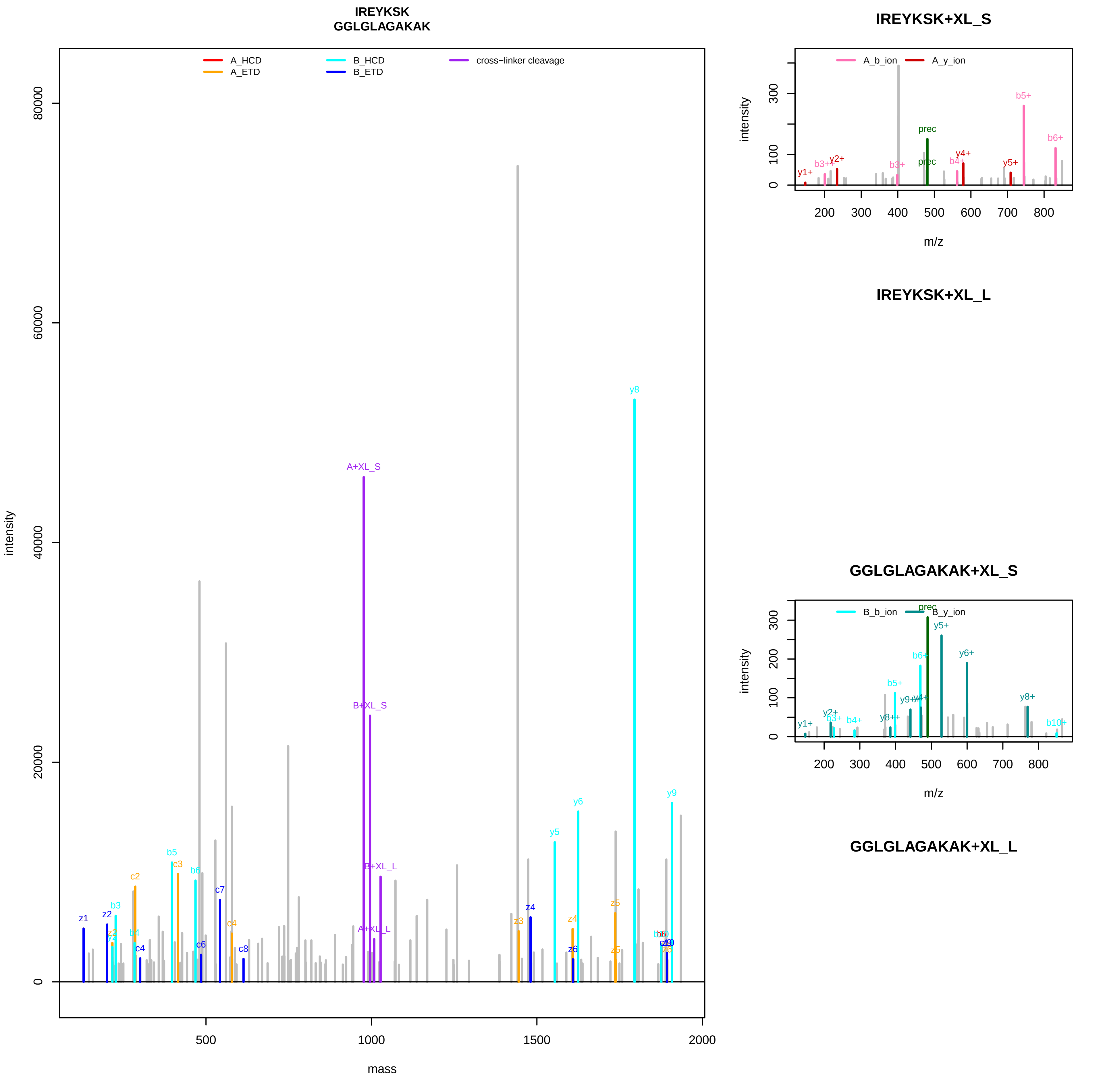
IREYKSK
GGLGLAGAKAK
A_HCD
A_ETD
B_HCD
B_ETD
cross−linker cleavage
intensity
mass
0
20000
40000
60000
80000
500
1000
1500
2000
z1
z2
b3
c2
b5
c3
b6
c7
c4
c6
c8
A+XL_S
B+XL_S
B+XL_L
A+XL_L
z3
z4
z6
y5
y6
y8
y9
z5
IREYKSK+XL_S
A_b_ion
A_y_ion
y1+
b3++
y2+
b3+
prec
b4+
y4+
y5+
b5+
b6+
m/z
IREYKSK+XL_L
GGLGLAGAKAK+XL_S
B_b_ion
B_y_ion
y2+
b3+
b4+
y8++
b5+
y9++
y4+
b6+
y5+
y6+
y8+
b10+
GGLGLAGAKAK+XL_L
